# Supplementary figures and images for: A functional bacteria-derived restriction modification system in the mitochondrion of a heterotrophic protist
Source: PLoS Biol. 2021 Apr 23;19(4):e3001126. doi: 10.1371/journal.pbio.3001126 (PMC8099122; doi:10.1371/journal.pbio.3001126)

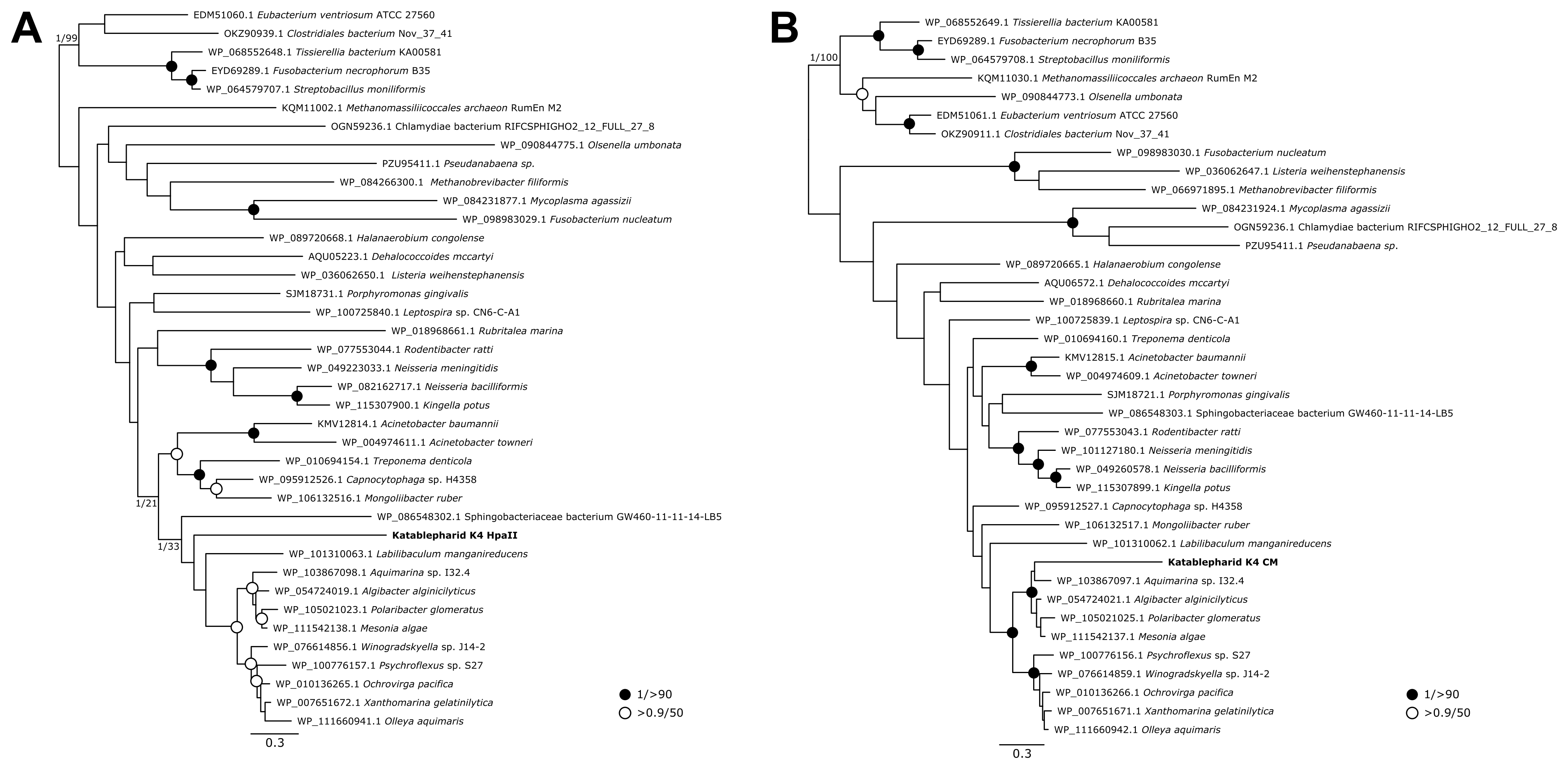

Supplement: S1 Fig — Phylogenetic reconstruction of HpaII (A) and HpaII-CM (B) encoded by the katablepharid mitochondrial genome. Phylogenies were reconstructed using sequences from K4 and 38 prokaryotic species containing tandemly encoded HpaII and HpaII-CM proteins, resulting in alignments of 352 and 415 positions, respectively. Support values are posterior probabilities calculated using MrBayes v3.2.6 [34] and 100 bootstrap replicates using RAxML v8.2.10 [35] and reported as MrBayes/RAxML. The MrBayes topology is shown. Bipartitions with support lower than 0.9/50 are unlabelled. Alignment data available at http://doi.org/10.6084/m9.figshare.c.5336963. CM, cytosine methyltransferase. (TIFF) [file pbio.3001126.s001.tiff]

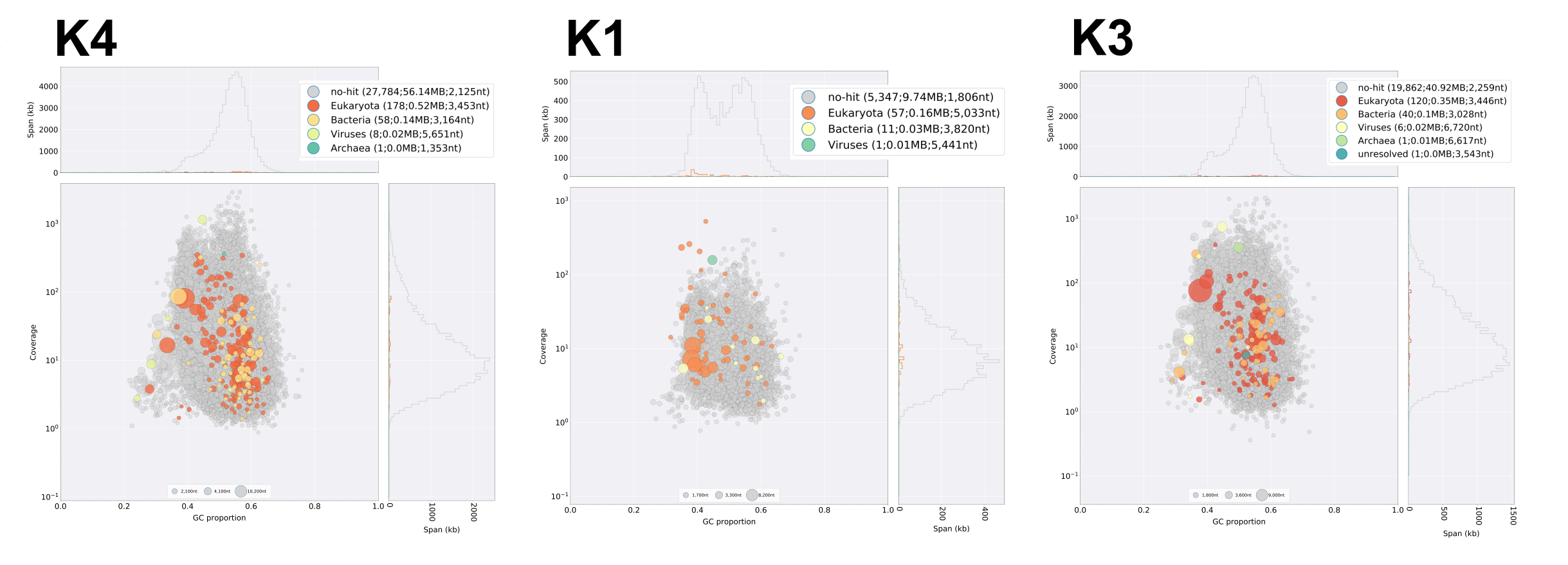

Supplement: S2 Fig — The contigs assigned to bacteria were low; as such, we have shown assignment only at the taxonomic level of “Bacteria” and have not shown lower taxonomic divisions. Blob-plots were generated using BLOBTOOLS [67] for the 3 SAGs (K4, K1, and K3) that mapped to katablepharids using contigs >1,000-bp. None of the contigs with best BLAST hits to bacteria were related to flavobacterial sequences. SAG contig data can be found at https://doi.org/10.6084/m9.figshare.7352966. SAG, single-cell amplified genome. (TIFF) [file pbio.3001126.s002.tiff]

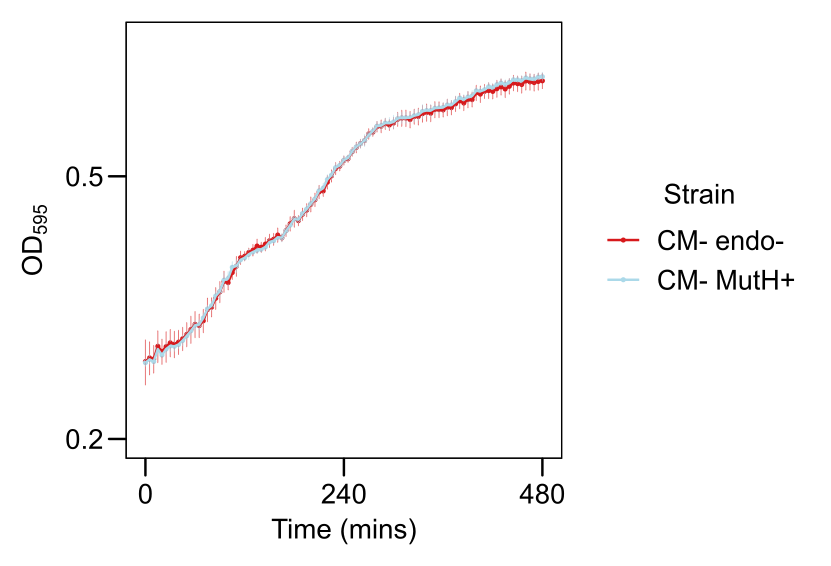

Supplement: S3 Fig — Growth of E. coli Top10 cells with pBAD plasmid containing putative MutH-like endonuclease (MutH) genes, or the corresponding empty vector. Cultures from three independent transformants were grown at 37°C for eight hours under Amp/Cm selection, induced with 0.1% arabinose, and growth was assessed by measuring OD595 at 5-minute intervals. This demonstrates that addition of the MutH-like endonuclease does not cause E. coli toxicity. Error bars represent one standard deviation from the mean. Underlying data in S6 Data. (TIFF) [file pbio.3001126.s003.tiff]

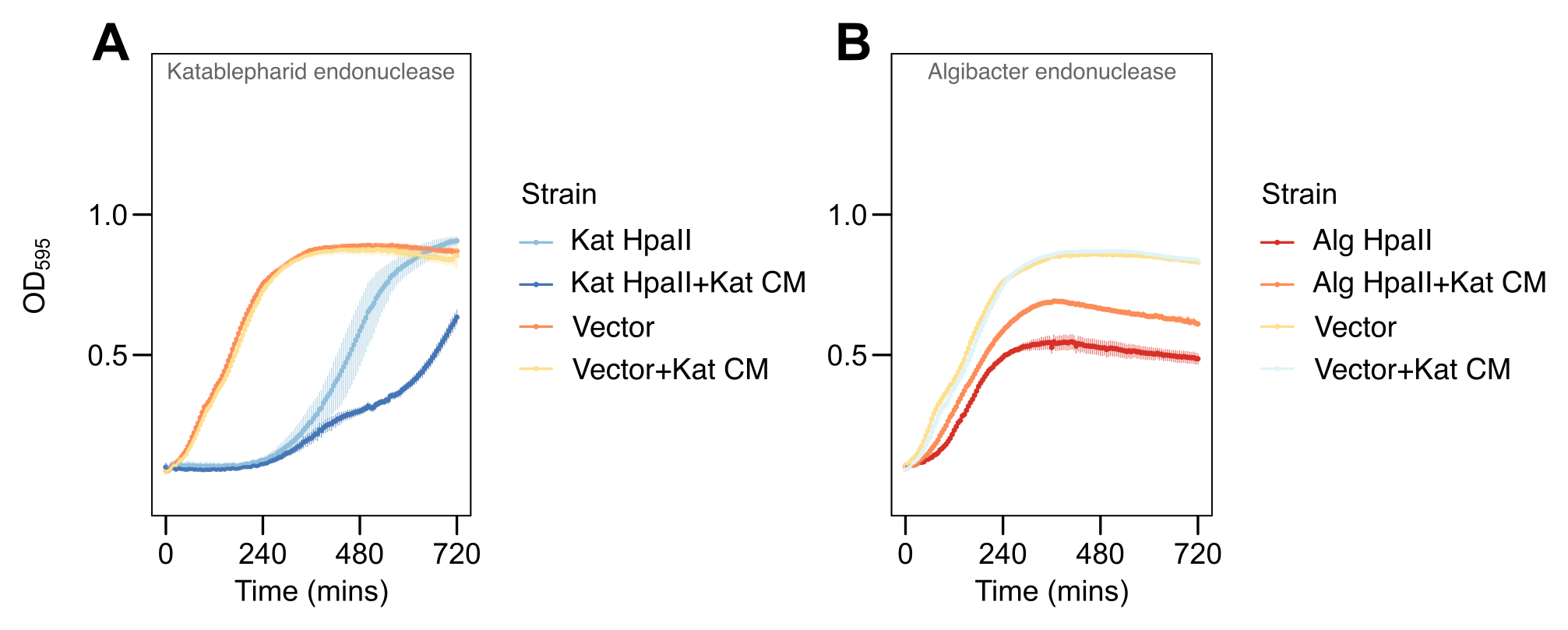

Supplement: S4 Fig — Removal of the start codon of the ORF from the endonuclease pBAD expression vectors (leaving only the start codon encoded by the vector) resulted in the katablepharid HpaII-CM no longer offering protection against the katablepharid HpaII endonuclease (Kat HpaII) (A). However, the katablepharid HpaII-CM was still able to protect against the Algibacter HpaII endonuclease (Alg HpaII) (B), these results point towards a necessary concentration/function minimum requirement for rescue of katablepharid HpaII endonuclease. Error bars represent one standard deviation from the mean of three independent E. coli transformants. Underlying data in (A) S7 Data and (B) S8 Data. CM, cytosine methyltransferase; ORF, open reading frame. (TIFF) [file pbio.3001126.s004.tiff]
