# Supplementary material for: A functional bacteria-derived restriction modification system in the mitochondrion of a heterotrophic protist
Source: PLoS Biol. 2021 Apr 23;19(4):e3001126. doi: 10.1371/journal.pbio.3001126 (PMC8099122; doi:10.1371/journal.pbio.3001126)
Supplement: S1 Table — (PDF) [file pbio.3001126.s005.pdf]

S1 Table: Primers used in this study

Primer	Sequence (5'- 3')	Role
Atp9_F_upstream	CGTAGAAAATCAGAGGCGGC	Confirmation of RM selfish element in mitochondria
Atp9_F	ACGTAGGAGCAGGATTAGCAA	Confirmation of RM selfish element in mitochondria
Kat_F_2385	CGTTGGGATTAGTACCTTCCG	Confirmation of RM selfish element in mitochondria
Kat_F_3745	ACGCAAATCAGCAAGTGGTT	Confirmation of RM selfish element in mitochondria
Kat_R_1727	CGAGACTACCACGCCTCATA	Confirmation of RM selfish element in mitochondria
Kat_R_4022	TCACACCAACGACTAAAGCA	Confirmation of RM selfish element in mitochondria
rns_kat_mito_R2	CGTCCGCCTAAAACCTTTGT	Confirmation of RM selfish element in mitochondria
pACYC184_5mC_F	TAGTGGTGGTGAAATTTGATAGGATTATAA	Amplification of bisulfite-treated *E. coli* plasmid DNA (targeting CCGG sites)
pACYC184_5mC_R2	CAATTACCAATAACTACTACCAATAATACT	Amplification of bisulfite-treated *E. coli* plasmid DNA (targeting CCGG sites)
pACYC184_region2_5mC_F2	AAGATATGTAAAAGTATTATTGGTAGTAGT	Amplification of bisulfite-treated *E. coli* plasmid DNA (targeting GATC sites)
pACYC184_region2_5mC_R2	CCTACAACATCCAAAATAACAATACCAAAA	Amplification of bisulfite-treated *E. coli* plasmid DNA (targeting GATC sites)
cox1_bisulfite_F	ATATTAAAGTATTGTAGAGATTAAATGTGA	Amplification of bisulfite-treated *S. cerevisiae cox1* region
cox1_bisulfite_R	ATAAATATTACCATCTCCTTCAAATAATCC	Amplification of bisulfite-treated *S. cerevisiae cox1* region
