# Supplementary material for: A functional bacteria-derived restriction modification system in the mitochondrion of a heterotrophic protist
Source: PLoS Biol. 2021 Apr 23;19(4):e3001126. doi: 10.1371/journal.pbio.3001126 (PMC8099122; doi:10.1371/journal.pbio.3001126)
Supplement: S2 Table — Note that all functions described are putative and constructs are codon-optimised for expression in E. coli, unless otherwise stated. (PDF) [file pbio.3001126.s006.pdf]

S2 Table: Plasmids used in this study. Note that all functions described are putative and constructs are codon optimised for expression in *E. coli*, unless otherwise stated.

Plasmid	Description
pDM027	pBAD HisA + Katablepharid HpaII endonuclease ORF
pDM034	pBAD HisA + *Algibacter* HpaII endonuclease ORF
pDM029	pBAD HisA + Katablepharid MutH endonuclease ORF
pDM040	pACYC184 + *Katablepharid* HpaII methyltransferase ORF
pDM041	pACYC184 + *Algibacter* HpaII methyltransferase ORF
pDM042	pACYC184 + Katablepharid MutH-like methyltransferase ORF
pDM071	pYES-mtGFP + Katablepharid HpaII endonuclease ORF (*S. cerevisiae* codon optimised)
pDM072	pYX223-mtGFP + Katablepharid HpaII methyltransferase ORF (*S. cerevisiae* codon optimised)
pDM076	pBAD HisA + Katablepharid HpaII endonuclease ORF, with ORF start codon omitted
pDM077	pBAD HisA + *Algibacter* HpaII endonuclease ORF, with ORF start codon omitted
