# Supplementary material for: A functional bacteria-derived restriction modification system in the mitochondrion of a heterotrophic protist
Source: PLoS Biol. 2021 Apr 23;19(4):e3001126. doi: 10.1371/journal.pbio.3001126 (PMC8099122; doi:10.1371/journal.pbio.3001126)
Supplement: S3 Table — Amino acid sequences for all proteins used in this study. (PDF) [file pbio.3001126.s007.pdf]

S3 Table: Protein sequences. Amino acid sequences for all proteins used in this study.

**Protein name**	**Amino acid sequence**
Kat-HpaII	MLKEVWCGNKGEWSESYTFLKLLGTKKLSVVSTIDKSEVAVYPILSVGTMRRGSLEIINHVIRNDEVVVGEEADISRRSVLEFQKNAEILLERIKSSDGNFSVPG<br>VQDFLESVRYSSFKASSRSKKDIVVGLQGMNREPLGFSIKSRLGSPPTLVNASRATNFIFQVKNLPDCEIGRINSERRIKDRVGSVLSLGGEFEFVRVESSIFLNNL<br>VLIDSRLPEILSKLVYEYYLGNSDSKISSLVEAITNKNPLNYDNDASQEVYKYKVKRFLIEVTLGLVPSVIWTGRRDAAGCLFVKTDGEILCYSSYDRVAFESYLFDN<br>LKLETGSTSRHSFGSLYKGDGGSTFVNFNLQIRFI*
Kat-HpaII-CM	MQEKVKLVYTTPKGYFYKITLDDRKKRVGRKEFLFLEACFKSVGVFVAPKKPEFKFVDLFAGIGGFRLALQNLKGECVFSSEWDKYSKQTYRENFGEIPFGDIRE<br>KSIMSHMPSGVDILCAGFPCQTFSSAGKRDGFEDTRGTLFFNVAEIVKETRPKAILLENVKGLRTHDKGKTLATMLKVLREDLRYFVPEPKILNSKDFGIPQNRE<br>RIFIVGFREDLKIREFEYPKGYTKKVNIKTILESEAVSVKYYLSEGYLSTLRNHKAKHRAKGSGFGYEIIPNDGIANTIMCGGMGREKNLLSDFRLKDFTPITRISGE<br>VNKEGIRKMTPREWARLQGFPDNYKIVVSDAQAYKQFGNAVTVPVVQAIAEEMLKKVKC*
Kat-MutH	MDDKGGFGKMVEGCYFKYEPNSSENPDFKVAGLELKCSPLKILKNGEFRSKEKLIMNIIDYMEVHKETFDKSAFLRKNSHLLLVFYLHDKNLDLVDYPVKLVA<br>NWQYTRDDLEIIERDWETITQKIRNGQAHELTESDTLCLGATVKGSIALKSLRKQPFNVAEAKQRAYSLKQCYVNHIIWSIAQEKSFKETFGKSLEGYRKTWSN<br>KGRI*
Kat-MutH-CM	MQEKVKLVYTTPKGYFYKITLDDRKKRVGRKEFLSLEACFTSERDCGLSGVSAKFEKIKVVELFAGVGGFRLGLEKSGYEIVWSNQWEPSTKIQHASKVYEARF<br>GKENHSNEDINKVITRNVEEIPDHDLLVGGFPCQDYSVAATLRDSKGLKGKKGALWWSIHEILKNKKNKPKYLFLENVDRLLKSPAKQRGRDFAVMLQSLNN<br>LGYALEWRVINAAEYGMPQRRKRVFIIGYHKSTEVYKRLQKSKEINWLTEEGTIANAFPVLKTTSIQEVELKGSLVEIAANFNKNGKLSFFQNAGLFIKGKVFTT<br>KTKPKYDGENAVLADVLEQGEVISDFFIDENDKSKWEYLKGAKTIERKSASGFIYEYSEGGMIYPDSLSNASRTIITGEGGKSPSRFKHVIVSSKGLRRLTPIELERL<br>NMFPDNHTKLNGIPDVKRAFFMGNALVVGVIERIGNKLYKQIKKKGI*
Algibacter-HpaII<br>(WP_054724019)	MITGNKGEWSEIYTLFKLLGDKQLFLGNKDIEKLEGIVYPILRVLRTENNGDFEYSIQDEIILISGGEEILKIAISEFKEKAKFLLEKIKSSKERTFSVPEIEEFMKSINCL<br>SLKASSTAKTDITIVVHDQRTNQQPTLGFSIKSQLGSPSTLLNAGKTTNFIFKISNTKLSESDVEKVNSIDSRSKIMDRINSVLQSNGQFGFVKTERQIFSNNLILID<br>SKLPEILSQIVYEFYSSDKSSIVDLVDKTTLKNPLGFDISNEHKFYEYKIKRFLTDIALGMMPSKVWTGEYDATGGYLIVKENGDVLCYHIYNRNEFENYLFNNTK<br>LDTASSNRHDFGTIYKENGELYFKLNLQIRFIK*
Algibacter-HpaII-<br>CM<br>(WP_054724021)	MLLKEKISIENFEDKKFKIRLIESKKDTEAAFTHYLHNHRNGVSQFYKPDAIEHVKTLFEYKFPEENISTLAAEEALQYLIFQQENIPFPAPKNPEFKFIDLFAGIGGF<br>RLAFQNLKGKCVFTSEWDKYSKQTYRANFGEVPFGDITKKETKNYIPDGFDVLCAGFPCQAFSIAGKRGGFEDTRGTLFFDVAEIIKKKKPKAIFLENVKGLRSH<br>DKGKTLATILNVLREDLGYFVPEPQIVNAKEFGVPQNRERIFIVGFRKDLGITEFEYPKPIKKKVTLENILETETVSVKYYLSETYVNTLRNHKARHESKGNGFGYE<br>IIPNNGTANAVVCGGMGRERNLVYDFRLKDFTPVTNIRGEVNREGIRKMTPREWARLQGFPDNYKIVVSDAQAYKQFGNSVAVPAIQATAKKIIEKIKTL*
