# Supplementary material for: A functional bacteria-derived restriction modification system in the mitochondrion of a heterotrophic protist
Source: PLoS Biol. 2021 Apr 23;19(4):e3001126. doi: 10.1371/journal.pbio.3001126 (PMC8099122; doi:10.1371/journal.pbio.3001126)
Supplement: Attachment — Submitted filename: PLoS_responses_to_comments_TAR3x_DM2.docx [file pbio.3001126.s016.docx]

Dear Editors and Reviewers,

Thank you for the careful consideration of our manuscript and the constructive reviews. Please find listed below a point-by-point response to all the comments. Our responses are written in black font. The review provided is written in blue font. We have also numbered the responses for accessibility and cross referencing. Please note that line numbers relate to the manuscript document with ‘all markup’ enabled.

Dear Dr Richards,

Thank you very much for submitting your manuscript "A functional bacterial-derived restriction modification system in the mitochondrion of a heterotrophic protist" for consideration as a Discovery Report by PLOS Biology. As with all papers reviewed by the journal, yours was evaluated by the PLOS Biology editors as well as by an Academic Editor with relevant expertise and by four independent reviewers.

You'll see that all of the reviewers were intrigued by your findings and highly complimentary about the manuscript in general, but each has a number of requests that should be addressed before further consideration. Based on the reviews, we will probably accept this manuscript for publication, provided you satisfactorily address the remaining points raised by the reviewers. Please also make sure to address the following data and other policy-related requests.

IMPORTANT:

a) Please attend to the requests from the four reviewers.

Please find below a point-by-point response to all the comments.

b) We note that you say that you did not receive any specific funding for this work; can you confirm that this is the case?

Yes, all funding has been declared in the Acknowledgements. This has been updated and added, where viable, to the submission pro-forma.

c) Please address my Data Policy requests further down, i.e. provide the data that underlie the main and supplementary Figures, and cite the location of those data clearly in the respective legends.

As you address these items, please take this last chance to review your reference list to ensure that it is complete and correct. If you have cited papers that have been retracted, please include the rationale for doing so in the manuscript text, or remove these references and replace them with relevant current references. Any changes to the reference list should be mentioned in the cover letter that accompanies your revised manuscript.

We expect to receive your revised manuscript within two weeks, but would understand if it took a little longer in the current challenging circumstances.

To submit your revision, please go to <https://www.editorialmanager.com/pbiology/> and log in as an Author. Click the link labelled 'Submissions Needing Revision' to find your submission record. Your revised submission must include the following:
-  a cover letter that should detail your responses to any editorial requests, if applicable, and whether changes have been made to the reference list
-  a Response to Reviewers file that provides a detailed response to the reviewers' comments (if applicable)
-  a track-changes file indicating any changes that you have made to the manuscript. 

NOTE: If Supporting Information files are included with your article, note that these are not copyedited and will be published as they are submitted. Please ensure that these files are legible and of high quality (at least 300 dpi) in an easily accessible file format. For this reason, please be aware that any references listed in an SI file will not be indexed. For more information, see our Supporting Information guidelines:
<https://journals.plos.org/plosbiology/s/supporting-information>  

*Published Peer Review History*
Please note that you may have the opportunity to make the peer review history publicly available. The record will include editor decision letters (with reviews) and your responses to reviewer comments. If eligible, we will contact you to opt in or out. Please see here for more details:
<https://blogs.plos.org/plos/2019/05/plos-journals-now-open-for-published-peer-review/>

*Early Version*
Please note that an uncorrected proof of your manuscript will be published online ahead of the final version, unless you opted out when submitting your manuscript. If, for any reason, you do not want an earlier version of your manuscript published online, uncheck the box. Should you, your institution's press office or the journal office choose to press release your paper, you will automatically be opted out of early publication. We ask that you notify us as soon as possible if you or your institution is planning to press release the article.

*Protocols deposition*
To enhance the reproducibility of your results, we recommend that if applicable you deposit your laboratory protocols in protocols.io, where a protocol can be assigned its own identifier (DOI) such that it can be cited independently in the future. For instructions see: <https://journals.plos.org/plosbiology/s/submission-guidelines#loc-materials-and-methods> 

Please do not hesitate to contact me should you have any questions.

Sincerely,

Roli Roberts


Roland G Roberts, PhD,
Senior Editor,
rroberts@plos.org,
PLOS Biology

------------------------------------------------------------------------
DATA POLICY:

You may be aware of the PLOS Data Policy, which requires that all data be made available without restriction: <http://journals.plos.org/plosbiology/s/data-availability>. For more information, please also see this editorial: <http://dx.doi.org/10.1371/journal.pbio.1001797> 

Note that we do not require all raw data. Rather, we ask that all individual quantitative observations that underlie the data summarized in the figures and results of your paper be made available in one of the following forms:

1) Supplementary files (e.g., excel). Please ensure that all data files are uploaded as 'Supporting Information' and are invariably referred to (in the manuscript, figure legends, and the Description field when uploading your files) using the following format verbatim: S1 Data, S2 Data, etc. Multiple panels of a single or even several figures can be included as multiple sheets in one excel file that is saved using exactly the following convention: S1_Data.xlsx (using an underscore).

2) Deposition in a publicly available repository. Please also provide the accession code or a reviewer link so that we may view your data before publication. 

Regardless of the method selected, please ensure that you provide the individual numerical values that underlie the summary data displayed in the following figure panels as they are essential for readers to assess your analysis and to reproduce it: Figs 2A (alignments), 3BCD, S1 (alignments), S2, S3, S4AB. NOTE: the numerical data provided should include all replicates AND the way in which the plotted mean and errors were derived (it should not present only the mean/average values).

This has all been collated into S4_Data.xlsx. The alignments for Fig 2A and S1 are now available at <https://figshare.com/s/ef18734856a9250edbec> (currently a private link; will be made public upon acceptance). We have also added the amino acid sequences for all proteins to S3_Table.

IMPORTANT: Please also ensure that figure legends in your manuscript include information on where the underlying data can be found, and ensure your supplemental data file/s has a legend.

This information has been added to each respective figure legend.

Please ensure that your Data Statement in the submission system accurately describes where your data can be found.

This has been updated

 ------------------------------------------------------------------------
BLOT AND GEL REPORTING REQUIREMENTS:

For manuscripts submitted on or after 1st July 2019, we require the original, uncropped and minimally adjusted images supporting all blot and gel results reported in an article's figures or Supporting Information files. We will require these files before a manuscript can be accepted so please prepare and upload them now. Please carefully read our guidelines for how to prepare and upload this data: <https://journals.plos.org/plosbiology/s/figures#loc-blot-and-gel-reporting-requirements>

Not applicable, as we show no gel images.
------------------------------------------------------------------------
DATA NOT SHOWN?

- Please note that per journal policy, we do not allow the mention of "data not sown", "personal communication", "manuscript in preparation" or other references to data that is not publicly available or contained within this manuscript. Please either remove mention of these data or add figures presenting the results and the data underlying the figure(s).

Not applicable
------------------------------------------------------------------------

REVIEWERS' COMMENTS:


Reviewer #1:

This manuscript describes the presence of foreign genes in the mitochondrial genome a katablepharid protist, including an apparently functional restriction-modification system of bacterial origin. This represents a very unusual event in eukaryotic history that may provide insight into the acquisition and spread of selfish elements in mitochondrial genomes. Therefore, it has the potential to be of broad interest. I do not have experience with the functional characterization of restriction modification systems, but to my inexperienced eye, the transgenic work appeared to provide compelling evidence that the HpaII-like element and corresponding methyltransferase have retained their ancestral enzymatic activity inferred from sequence homology. Therefore, it is likely that they do constitute an intact restriction modification system in the protist. While I found the manuscript to be clearly presented and interesting, I do have some comments and concerns, which are listed below.

1.      I agree with the authors' framing of the RM system as a likely selfish element that would be capable of preferentially spreading in the context of heteroplasmy with other mitochondrial haplotypes. However, I do not see the rationale for the authors' speculation that such a system was co-opted in the evolution of uniparental inheritance (last sentence of abstract). Yes, an RM system would result in biased outcomes when biparental inheritance and mitochondrial fusion are occurring. But I do not see that being the same thing as uniparental inheritance, which to me implies that there is a regulated system resulting in the consistent transmission of the mtDNA from just one sex, mating type, etc. For example, note that if an RM system was successful in spreading to fixation in a population with biparental inheritance, there would no longer be any bias in inheritance during sexual reproduction. If anything, much of the relevant literature has focused on the hypothesis that
uniparental inheritance has evolved as a response to suppress such selfish elements. For these reasons, I would suggest revising the last sentence of the abstract and the paragraph starting on line 296 to focus on these as selfish genetic elements rather than a mechanism of uniparental inheritance.

Response 1.1: Based on this comment, and that of the other reviewers, we have removed the ‘uniparental inheritance’ aspect of this manuscript. We have focussed, as suggested, on this RM system as a selfish genetic element, and given their propensity to spread preferentially, we feel that the biased (i.e., rather than uniparental) organellar inheritance aspect of the manuscript is still a key discussion point, particularly in the context of a Discovery Report to galvanise further scientific advances in the field. We have changed the abstract to read: “These results suggest that a mitochondrial-encoded restriction modification system can function as a toxin-antitoxin selfish element, and that such elements could be co-opted by eukaryotic genomes to drive biased organellar inheritance.” We have also changed lines 358-402 (i.e., the last two paragraphs of the discussion) to reflect this focus on ‘biased’, rather than ‘uniparental’, organellar inheritance.

2.      Line 57-58. "no gain-of-function transfers into the mitochondrial genome have, to our knowledge, been previously reported." Perhaps I am misinterpreting the authors' meaning here, but I do not believe this is an accurate statement. What about the acquisition of mutS gene in the mitochondrial genomes of octocorals? Or the ORFs of likely viral origin in bivalves with doubly uniparental inheritance? There may be other examples, as this is not a topic I've ever tried to search out comprehensively in the literature or published mitogenomes. There are also other cases that may be relevant, though perhaps not directly contradictory to this statement. For example, there are many mitochondrial plasmids in plants, fungi, and ciliates that harbor a whole suite of foreign genes, many of which have been incorporated into mitochondrial chromosomes. Plant mitogenomes have also acquired many functional tRNAs from plastids and bacteria (perhaps some of the "replacement genes" that authors referred to). There is also at least one case where a nuclear-derived gene was shown to be transcribed in plant mitochondria (Qiu et al. 2014: <https://www.sciencedirect.com/science/article/pii/S2214662814000073>).

Response 1.2: We thank the reviewer for this point; we had aimed to portray that these genes are transcribed (based on our meta-transcriptomic data analysis), and, therefore, also likely to be functional in katablepharids, given our assessment of function. We agree that the phrasing of this point currently appears inaccurate. As such, we have edited this line to now read: “While novel functional genes have entered chloroplast genomes, and replacement genes can find their way into mitochondrial DNA (mtDNA), the scarcity of gain-of-function transfers identified within mitochondrial genomes makes them intriguing case studies for assessing the functional consequences of such gene acquisitions.” We have also added the mutS octocoral and bivalve viral genes references to line 57.

3.      Line 189. "Domestication" is a loaded term with a lot of implications. The fact that codon usage is evolving toward a higher degree of similarity with the mitogenome could reflect something as simple as it being exposed to the same mutation pressures because the RM genes are now located in that genome. I would suggest revising this sentence to avoid suggesting that the changes in codon usage are evidence of domestication.

Response 1.3: Please see also response 4.4; ‘domestication’ has been changed to ‘amelioration’ to avoid the additional implications associated with the word ‘domestication’

4.      Katablepharid protists are a lineage that will be unfamiliar to most readers (including me). A little more content in the introduction on what they are and where they fall within the tree of life might be helpful.

Response 1.4: This information has been added: “a group of flagellated heterotrophic unicellular protists, approximately 10 µm in length, that are found in both marine and freshwater environments” (line 96-98). We have also added three references to support this addition (Vørs 1992, Auer & Arndt 2001 and Clay & Kugrens 1999).

5.      Whereas other figures indicate that error bars represent one StdDev, this information in not provided in Fig S4. In addition to adding that information to the figure legend, I suggest more generally clarifying the extent to which replicates in these analyses are (non)independent. For example, are they performed with independent transformants or splitting of a single transformed line?

Response 1.5: We apologise that this StdDev information was omitted; this has now been added to the legend for Figure S4 (line 882-883). We have also included the information on replicates - to state that all replicates were based on independent transformants - for each experiment.

Reviewer #2:

This very nice manuscript by Milner and colleagues describes the horizontal transfer of a bacterial restriction modification system into the mitochondrial genome of a protist. The authors perform functional experiments to demonstrate that the transferred genes are functional and propose a hypothesis for how the acquisition of this restriction modification system may be behaving as a selfish genetic element.

The work is quite novel and beautifully done. The manuscript is clearly written and will be of great interest to the audience of PLoS Biology and of evolutionary biologists in general. I only have a few minor comments for the authors to consider:

We thank the reviewer for this positive assessment of our work

- lines 36-38: what do the transferred genes have to do with uniparental inheritance is not at all clear from the abstract. Part of what's missing is a clear statement that the restriction modification system is a type of selfish element - the other part is explaining more the connection between selfishness and uniparental inheritance. The authors have explained these connections in the main text but not in the abstract.

Response 2.1: We have reworded the last sentence of the abstract to make a clear statement that the RM system can function as a (toxin-antitoxin) selfish element. As indicated in response 1.1, we have removed the link between the selfish element and uniparental inheritance, instead focussing on the possibility to promote ‘biased’ organelle inheritance

- line 59" "Truly 'selfish' elements". What do you mean by "truly"? Are there selfish elements that are not truly selfish? and why the quotation marks for such a standard term?

Response 2.2: We agree that this adds unnecessary complexity to this section. This has been rephrased to remove ‘truly’ and the quotation marks

- paragraph starting on line 111: in this paragraph, i would have appreciated some additional info on the taxonomic provenance of the taxa mentioned (e.g., Algibacter belongs to Flavobacteria). Whether the reported identity scores are at the amino acid or nucleotide level also needs to be reported

Response 2.3: We have clarified that this is amino acid identity (lines 143-151) and added the taxonomic provenance of the taxa mentioned in this section.

- line 148: "strong nucleotide identity" -> "high nucleotide identity"

This change has been made (line 185): “high percentage nucleotide identity”

- line 165: "limited bootstrap support" -> "low bootstrap support"

This sentence has been changed to “The phylogeny of the restriction endonuclease showed the mitochondrial genes branching basally to the flavobacteria, with weak bootstrap support” to reflect this suggestion

- line 176: "relatively extended branch" -> "long / longer branch"

This change has been made to “longer”

- lines 177-8: i get how positive or relaxed selection could result in a long branch, but I did not understand how population bottlenecking could lead to (such) a long branch

Response 2.4: We have removed this point on population bottlenecking

- line 78, 262, and possibly elsewhere: Not sure what the policy of PLoS Biology is, but i generally find claims of priority ("the first known instance detected within a eukaryotic genome") not particularly helpful. I leave it up to the authors, but my advice would be to remove them from the manuscript and instead describe what their novel finding is and its significance.

Response 2.5: We have removed both of these claims of novelty

Reviewer #3:

This is a very interesting paper presenting compelling evidence for there being a restriction modification system (which are usually found in bacteria) in the mitochondria of a eukaryote (specifically, a katablepharid protist). I only have a few minor comments:

Abstract:

line 33: the connection to the flavobacteria is not overly compelling, as the phylogeny shows a long branch that connects pretty basally in the phylogeny, and I would suggest rewording to more accurately report the observation -- that phylogenetic analysis with known databases puts the closest relatives of the genes in the flavobacteria.

Response 3.1: We agree that this is not clear at points in the manuscript. As such, we have updated line 207-209 to “The phylogeny of the restriction endonuclease showed the mitochondrial genes branching basally to the flavobacteria, with weak bootstrap support”. Given that there is strong bootstrap support for the placement of the mitochondrial RM system with the flavobacteria in the concatenated alignment (Fig 2a) and the Kat-HpaII-CM (Fig S1b), we feel that this is accurately represented elsewhere in the manuscript. We feel that line 33 (now line 35) accurately reflects the nature of the phylogeny “…involving bacteria *related to* flavobacteria”, particularly given that the BLASTP REBASE analysis suggests that 3/4 of the RM components have top hits in the flavobacteria (see point 2.3: we have also updated this section to reiterate this point).

lines 37-8: there is very little in the paper about the possible co-option of RM systems by the nucleus to drive uniparental inheritance, and either this sentence should be deleted or modified, or the ideas fleshed out in the discussion. As it is, there is not much evidence presented for this (the Chlamydomonas work being ambiguous), and it seems a shame to end the abstract on such a dubious note, rather than focussing on the novel class of mitochondrial selfish elements.

Response 3.2: Based on this comment, and that of the other reviewers, we have removed the ‘uniparental inheritance’ aspect of this manuscript (see response 1.1). Instead, this line in the abstract reads “These results suggest that a mitochondrial-encoded restriction modification system can function as a toxin-antitoxin selfish element, and that such elements could be co-opted by eukaryotic genomes to drive biased organellar inheritance.” Given our finding that the endonuclease can degrade mitochondrial DNA (Figure 4C), we feel that this is an important discussion point that will hopefully prime further work in how selfish genetic elements may affect/bias mitochondrial inheritance, hence the submission of this paper as a ‘Discovery Report’.

line 174: What is meant by 'recent'? Can you rule out 100million years? 500 million? And can you rule out the possibility that there were 1 or more intermediary species between the flavobacterium ancestor and your protist that are not yet in the databases?

Response 3.3: We have changed the phrasing of this point to remove the word ‘recent’. This somewhat reveals our bias, as we think on evolution on the scale of domains of life, so everything is recent for us. We apologise for this oversight.

lines 268-271: be more explicit about the degeneration seen.

Response 3.4: This has been added: “including loss of the HpaII/HpaII-CM genes, and presence of a partial MutH-CM gene,” (line 341-342)

lines 297-300: This sentence does not make sense to me. Differential parental inheritance will occur while the RM system is rare in the population, but then when it is common and everyone has it, then there is no more differential inheritance -- it is a purely transient thing. Given that, it is not clear how important it is?

Response 3.5: We agree that this would be a transient process which - in theory - would drive the selfish element to fixation. However, for marine protists, i) population stratification and ii) sexual reproduction is likely not obligatory, suggesting that mtDNA-encoded selfish elements would be unlikely to reach 100% fixation. We also note, however, that there was degeneration of the RM element in some of the katablepharid samples (Fig 1B). This would be consistent with the possibility that, at least at that sampled geographic site and/or time, the element may have reached fixation and was then subject to relaxed selection and degeneration.

This discussion paragraph suggests that this process *could* happen in katablepharids, given that they harbour genes which may allow them to be capable of sexual reproduction (something which has not previously been reported for this group). We have changed the lead sentence in this paragraph to iterate that this is something that *could* be driven by the selfish element, rather than something that *is* happening *per se* (line 358: copied below). We feel that this point is important to consider. Further, we have amended a number of sections where we discuss ‘uniparental’ inheritance, instead focussing on the potential to lead to biased organellar inheritance (lines 40 & 365). We feel that this change solves the main point of the reviewer here.

Line 358: “Third, and most intriguing among these possibilities, this RM element could act to bias spread of the host mtDNA within the katablepharid population.”

Reviewer #4:

Review of Milner et al. Plos Biology 2021

The paper describes a case of horizontal gene transfer of a two-gene module the authors later go on to confirm is involved in methylation and degradation of DNA with precise motifs. Authors infer that this HGT results in mitochondrial drive.

This is an exceptionally well-written manuscript, which takes a very simple observation of anomalous sequence in a genome and successfully tests a number inferences about the significance of its existence. The work is boosted by tests at multiple scales, e.g. expression of the sequence in global metagenomes, heterologous expression/characterization of the sequence and its pre-HGT bacterial counterpart, imaging of co-location of target DNA and enzyme expression, codon evolution, etc. There are a number of broadly interesting outcomes, including exceptional events in molecular evolution (expansion of organelle genome), cooperation between selfish genetic elements and host, and a novel mechanism of the origin of genetic drive.

We thank the reviewer for their positive comments.

I only have a couple of comments.

"SAG" was not intuitive in the figure without reading multiple points of the text. Figure 1 especially would be much more readily grasped with an indication what SAG means/implies. Either spelling out the phrase, or adding a modifier to the mitochondrial genome names that indicates what is being distinguished by the re-sequencings.

Response 4.1: This definition has been added to the legend of Figure 1

Along with this, there is room for misinterpretation in line 140-141. The sentence is describing the reproducibility, but could be interpreted to mean independent evolutionary events. I think this would be avoided by stating the result more plainly and without combining with the inference. Something more like "amplification of the bacteria-like RM systems from mitochondrial genomes was independently replicated 5 times."

Response 4.2: Thank you for noticing this potential ambiguity; we have amended this section as suggested (line 177): “In total, amplification of the bacteria-like RM systems from mitochondrial genomes was independently sampled 5 times.”

The legend in Figure 2A suggests Bayesian posterior probabilities can exceed 1 ("black dot > 1/90"). This should be more clear.

Response 4.3: We have updated this to “1/>90” to avoid this. We have also made this change in Figure S1

The "domestication" referred to in line 189 has the specific term "amelioration" assigned to it. I am not sure which term brings more unnecessary implication, but the latter would be better connected to the appropriate literature.

Response 4.4: We agree that the word domestication suggested additional implications in terms of protein function. As such, we have changed this word to ‘amelioration’, as suggested. This change indicates that the sequence is changing towards that of the mitochondrial genome, but does not have the functional implications associated with ‘domestication’
